# Near Infrared Spectroscopy (NIRS) as a New Non-Invasive Tool to Detect Oxidative Skeletal Muscle Impairment in Children Survived to Acute Lymphoblastic Leukaemia

**DOI:** 10.1371/journal.pone.0099282

**Published:** 2014-06-23

**Authors:** Francesca Lanfranconi, Luca Pollastri, Alessandra Ferri, Donatella Fraschini, Giuseppe Masera, Giuseppe Miserocchi

**Affiliations:** 1 Department of Health Sciences, Laboratory of Clinical Physiology and Sport Medicine, University of Milano-Bicocca, Monza, Italy; 2 Department of Pediatrics, University of Milano-Bicocca, San Gerardo Hospital, Monza, Italy; Universidad Pablo de Olavide, Centro Andaluz de Biología del Desarrollo-CSIC, Spain

## Abstract

**Background:**

Separating out the effects of cancer and treatment between central and peripheral components of the O_2_ delivery chain should be of interest to clinicians for longitudinal evaluation of potential functional impairment in order to set appropriate individually tailored training/rehabilitation programmes. We propose a non-invasive method (NIRS, near infrared spectroscopy) to be used in routine clinical practice to evaluate a potential impairment of skeletal muscle oxidative capacity during exercise in children previously diagnosed with acute lymphoblastic leukaemia (ALL). The purpose of this study was to evaluate the capacity of skeletal muscle to extract O_2_ in 10 children diagnosed with ALL, 1 year after the end of malignancy treatment, compared to a control group matched for gender and age (mean±SD = 7.8±1.5 and 7.3±1.4 years, respectively).

**Methods and Findings:**

Participants underwent an incremental exercise test on a treadmill until exhaustion. Oxygen uptake (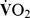), heart rate (HR), and tissue oxygenation status (Δ[HHb]) of the *vastus lateralis* muscle evaluated by NIRS, were measured. The results showed that, in children with ALL, a significant linear regression was found by plotting 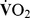 *vs* Δ[HHb] both measured at peak of exercise. In children with ALL, the slope of the HR *vs* 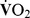 linear response (during sub-maximal and peak work rates) was negatively correlated with the peak value of Δ[HHb].

**Conclusions:**

The present study proves that the NIRS technique allows us to identify large inter-individual differences in levels of impairment in muscle O_2_ extraction in children with ALL. The outcome of these findings is variable and may reflect either muscle atrophy due to lack of use or, in the most severe cases, an undiagnosed myopathy.

## Introduction

Approximately 1500 children under 15 years of age are diagnosed with cancer each year in Italy, of which acute lymphoblastic leukaemia (ALL) is the most common malignancy accounting for 38% of all childhood cancers [1]. In the last few decades progress in treatment has considerably increased the survival rates of children with ALL and a 70% cure rate for children with standard-risk disease is provided by the Italian Association of Paediatric Haematology and Oncology (*AIEOP, Associazione Italiana Emato Oncologia Pediatrica*) [2], [3]. It has been estimated that 1 out of 900 persons (aged 18–34 years), has been treated for cancer during their childhood [3], [4]. Among the potential consequences of childhood cancer therapy are death, neuro-psychiatric adverse events and myopathy, pain, fatigue, obesity, osteoporosis, cardiomyopathy and neuromuscular complications [5]–[8]. Therefore, in order to better understand the consequences of ALL or malignancy treatments, a long-term follow up in a large number of young people will assist to evaluate the resiliency and health-related quality of life [3], [4].

The main system of energy supply within cells is *via* oxidative metabolism. Any impairment along the “O_2_ transport and utilization chain” significantly affects skeletal muscle function and exercise capacity. Peak O_2_ uptake (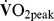), considered as an individual index of aerobic fitness, was found to be reduced in children with ALL because malignancy treatments can lead to chronic pulmonary disease [9] and/or to cardiovascular impairment [10], [11]. The reduced 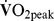 in children with ALL has been attributed principally to the inability of the cardiovascular system to deliver O_2_ to skeletal muscles (the “central” component of the O_2_ transport chain) [5]. However, restricting the focus solely to O_2_ delivery may not be appropriate and could cloud understanding of the physiological basis of aerobic fitness as originally proposed by the Fick equation [12]. Indeed, muscle O_2_ extraction in children with ALL might be damaged both by a decrease in O_2_ delivery as well as by an impairment in O_2_ tissue utilization (a “peripheral” component of the O_2_ transport chain). Some authors have suggested that the reduced exercise capacity in children with ALL might also reflect drug-induced side effects such as myopathy, osteonecrosis (often asymptomatic), impairment of the peripheral nervous system, and/or a decrease in skeletal muscle oxidative metabolism [5], [8], [13]–[17]. Furthermore, a sedentary life style could also affect the aerobic fitness of this population [5]: a lower level of functioning of the skeletal muscle pump and of the vasodilatatory capacity, as seen in unfit persons, could affect the ability of tissue O_2_ extraction due to malignancy treatment [12].

Although several studies have reported muscle atrophy in long-term survivors of ALL, there is a paucity of data regarding the time-course of functional impairment in children with ALL. Following up on metabolic impairments along the O_2_ transport utilization chain, after ALL treatment, or on the effect of specific rehabilitation programmes, a non-invasive evaluation approach is desirable. This could be obtained by near infrared spectroscopy (NIRS), a non-invasive methodology which provides an estimate of O_2_ utilization of the peripheral muscles [18], [19]. Oxygenation NIRS indices are the result of the balance between O_2_ delivery and muscle 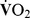 in the portion of tissue under consideration, being therefore conceptually similar to muscle O_2_ extraction [20]. The changes in NIRS light absorption in working muscles reflect adjustments in oxygenation at the level of the microcirculation and intracellular sites of O_2_ transport and uptake. More specifically, for a given 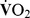, a decrease in O_2_ extraction (deoxygenation) relative to O_2_ delivery decreases the NIRS signal, while the opposite is true for an increase in O_2_ extraction: therefore this technique has been previously utilized to evaluate the efficiency of the peripheral component of aerobic fitness [18]–[20].

The purpose of this study was to evaluate non-invasively the potential limitations of the O_2_ transport and utilization chain in children with ALL. We contend that an evaluation of the efficiency of the central and peripheral components of 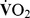 in children with ALL (above and beyond the information provided by 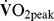), extending from the onset of malignancy treatment through to long term follow up, would be of particular interest considering that: 1) a decreased exercise capacity could be related to muscle atrophy due to lack of use or, in the most severe cases, has to raise suspicions about an underlying metabolic myopathy; 2) rehabilitation and training programmes are specifically focused on skeletal muscle reconditioning. Our study hypothesized that NIRS would allow us to identify and quantify any impairment of muscle O_2_ extraction during an incremental exercise test, especially at submaximal workloads. Indirect evidence in favour of this hypothesis would derive from the observation of significant correlations between the NIRS-derived index of O_2_ extraction, 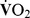 and the extent of cardiovascular response to submaximal exercise.

## Materials and Methods

### Participants

Children with ALL (n = 10) were compared to an equal number of healthy children (CTRL), the latter group recruited as peers from different schools in the same geographical area, matched for age (mean ± standard deviation (SD) = 7.8±1.5 and 7.3±1.4 years, respectively), gender and sexual maturity. Sexual maturity was assessed by medical doctors using the indices of pubic hair and male genital or female breast development as described by Tanner (stage 1, 80% and stage 2 20% of participants in each group). Experienced medical doctors specialized in sports medicine collected details concerning the physical activities reported by children (daily life, leisure activities and recreational actvities). All the children were at limit for being considered physically active as defined by the 2008 physical activities guidelines for Americans: they performed recreational activities (aerobic, muscle-strengthening and bone-strengthening exercise) less than 3 days a week, these activities were appropriate and enjoyable for their age and did not require experience of exhaustive exercise. The clinical characteristics and weekly recreational activities performed by children with ALL as well as CTRL, are shown in Table 1. Inclusion criteria were absence of anaemia, neuropathy, orthopaedic, pulmonary and/or cardiovascular disorders. There were no exclusions on medical ground for infective diseases. Every attempt to avoid any unnecessary discomfort and disturbance to all participants and parents was made according to the ERICE statement about the cure and care of long-term survivors of childhood cancer [21]. All children and their parent(s) provided informed assent and written consent to participate in the project, which was approved by the University of Milano Bicocca (Milano – Italy) ethics committee. Personal data was treated according to standard principles of confidentiality.

**Table 1:** Clinical characteristics and recreational activities of children with acute lymphoblastic leukemia (ALL) and controls (CTRL).

ALL	Gender	Age	Risk factor	Time elapsed since diagnosis of ALL	Clinical events during maintenance therapy	Recreational activities
		*yrs*		*months*		*minutes, weekly, last 6 months*
A1	M	10	S	31	none	swimming 50′×2
A2	M	6	I	32	none	soccer 50′×2
A3	F	10	I	37	none	swimming 50′
A4	M	8	H	34	none	/
A5	F	8	S	43	none	swimming 50′
A6	M	7	I	30	none	soccer 70′
A7	M	6	S	35	none	judo 50′
A8	M	6	I	41	none	swimming 50′×2
A9	F	8	S	35	Leishman.	kick boxing 70′
A10	M	9	I	35	none	soccer 70′×2
**CTRL**						
C1	M	7				climbing 70′, swimming 50′
C2	F	9				volleyball 50′, swimming 50′
C3	F	6				artistic gymnastic 50′×2
C4	M	9				athletics 70′
C5	F	7				swimming 50′×2
C6	M	7				savate 50′
C7	F	6				swimming 50′
C8	M	6				savate 50
C9	M	6				savate 50′
C10	M	10				karate 50′×2

*S = standard risk; I = intermediate risk; H = high risk (see text for further explanation).*

### Risk group definitions and final stratification

Children with ALL, all consecutive clinical attendees, came to the laboratory after attending their routine follow up, for a complete physical examination by the paediatricians at the Department of Paediatrics, S. Gerardo Hospital (Monza –Italy). To stratify ALL patients in prospective studies, a standardized quantitative assessment of minimal residual disease (MRD), based on immunoglobulin and T-cell receptor gene rearrangements as polymerase chain reaction targets at 2 time points, was introduced by AIEOP and the Berlin Frankfurt Munster (BFM) ALL study in 2000 [3]. The oncologists of the Department of Paediatrics classified patients with precursor B ALL in MRD as standard risk (MRD-SR) if MRD was already negative at day 33 (analyzed by 2 markers, with a sensitivity of at least 10^−4^), as MRD high risk (MRD-HR) if MRD was 10^−3^ or more at day 78 and as MRD intermediate risk (MRD-IR) for all the others.

Our MRD-SR patients were 41%, MRD-IR were 50% and 1 patient was defined MRD-HR. The time period elapsed from the end of treatment in children with ALL, against MRD–SR, MRD–IR and MRD-HR following the AIEOP BFM ALL 2000 study protocol, until the exercise test session was approximately 1 year (11.3±4.3 months, range 6–20 months). One patient received cranial irradiation for MRD-HR.

### AIEOP-BFM ALL 2000 Treatment protocol: induction and consolidation phase

The backbone of contemporary multiagent chemotherapeutic regimens is formed by 4 elements: induction, central nervous system (CNS)-directed treatment and consolidation, reinduction and maintenance. All patients underwent 7 days of prephase with steroid therapy (prednisone) and 1 intrathecal dose of methotrexate (intrathecal MTX), followed by induction phase IA and induction consolidation phase IB; from day 8, patients were randomized to continue steroid treatment with either prednisone (60 mg (m^2^)^−1^ per day) or dexamethasone (10 mg (m^2^)^−1^ per day) until day 28 with subsequent tapering of dose within a week; ***Protocol M and reinduction phases*** *.* MRD-SR and IR patients received 4 cycles of high-dose MTX (5 g (m^2^)^−1^); at the beginning of the reinduction phase, patients were randomized to receive either protocol II or reduced-intensity protocol III in MRD-SR group, or protocol II versus reduced-intensity protocol III given twice in the MRD-IR group. MRD-HR patient were randomized to receive 3 blocks of non–cross-resistant drugs followed by protocol III given 3 times versus 3 blocks followed by protocol II given twice in the AIEOP group; ***Maintenance therapy***. Maintenance therapy consisted of daily 6-mercaptopurine together with weekly MTX until 24 months after diagnosis; ***CNS-directed therapy***. CNS-directed therapy consisted of repeated intrathecal MTX administration during each treatment phase. Cranial radiotherapy was given (dosage by age) to patients at MRD-HR or with CNS involvement at diagnosis.

### Exercise protocol

Before starting the test session individual body mass index (BMI) was calculated for children and was plotted on the American Academy of Pediatrics BMI for-age growth charts to obtain a percentile ranking. All exercise tests were conducted under close medical supervision and with 12-lead electrocardiography monitoring (Quark C12x: Cosmed, Roma, Italy). Children were allowed time to gain familiarity with the researchers and the experimental protocol by means of short preliminary practice runs on a treadmill. Parents were allowed to be present during the test session. A careful assessment that the participants were in a resting condition was carried out before the tests by measuring heart rate (HR), pulmonary ventilation (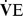, in BTPS) and observation of the child. An incremental exercise test was performed on a treadmill (Pulsar: h/p/Cosmos, Dusseldorf, Germany). After 2 minutes of walking at 1.5 km h^−1^ at 0% gradient, a constant-load exercise was conducted at 2.5 km h^−1^ at 5% slope for 4 minutes, thereafter the speed and gradient were increased by 0.1 km h^−1^ and 0.5% every 30 seconds until voluntary exhaustion was reached. Peak efforts were considered to have been given if, in addition to subjective indications, such as sweating, hyperpnea, and facial flushing, there was a consistent reduction in walking cadence, despite strong verbal encouragement. The exercise was stopped by the supervisor on the basis of subjective exhaustion feeling showed by the participant. The peak work rate was defined as the work rate attained at the point of test termination [11]. Uphill running offers the chance to estimate the external mechanical work done by participants to increase the potential energy of the body calculated by W = weight*ΔH, where ΔH is the vertical distance given by ΔH = D*sinα, where D is the distance run on the incline and α is the angle between zero level and the treadmill inclination. The average duration of the treadmill test for children with ALL and CTRL was 10.0±0.8 and 10.1±2.5 minutes respectively. 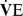, 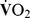, and CO_2_ output (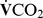) were determined breath-by-breath by a computerized metabolic cart (Vmax SPECTRA 229: SensorMedics Corporation Yorba Linda, California, USA). Expiratory flow was determined by a mass flow sensor (hot wire anemometer). 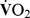 and 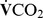 were determined through continuous monitoring of PO_2_ and PCO_2_ at the mouth throughout the respiratory cycle and from established mass balance equations. Ventilatory efficiency was estimated by the slope of 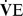 *vs* 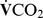 below the ventilatory compensation point for exercise metabolic acidosis [22]. HR was determined from a 12-lead electrocardiographic signal interfaced to SensorMedics metabolic cart. Arterial blood O_2_ saturation (SaO_2_) was monitored continuously through pulse oximetry at the finger (RAD 9 Signal Extraction Pulse Oximeter: Masimo Corporation, Irvine - California, USA). The environmental temperature during exercise was standardised to 20°C using an air-conditioning system and the current barometric pressure was recorded. The rated perceived exertion (RPE) was collected through the Borg scale aid.

### NIRS technique

NIRS is a non-invasive method that allows the monitoring of muscle oxygenation on the principle that the near-infrared (NIR) light absorption characteristics of haemoglobin (Hb) and myoglobin (Mb) depend on their O_2_ saturation. The absorption characteristics of light at 780 and 850 nm depend on the relative oxygenation of Hb and Mb. Mb has similar absorption spectra to Hb. In human skeletal muscle, however, the ratio [Hb]/[Mb] is higher than 5.19 so the signal is usually considered as deriving mainly from Hb. In view of these facts, it is appropriate to refer to NIRS measurements as indices of overall tissue oxygenation, with the understanding that ∼70% of underlying vascular beds are composed of venous blood. In the present study data were expressed as a variation of absolute units, Δ[O_2_Hb] and Δ[HHb], from the baseline value recorded after a 2 minutes light warm up and as a function of time. Therefore, decreases in Δ[O_2_Hb] and corresponding increases in Δ[HHb] were interpreted as evidence of relative muscle deoxygenation and, conversely, as evidence of improved oxygenation [19]; [20]. Since Δ[HHb] is closely associated with changes in venous O_2_ content, it is believed to be a sensitive measure of relative tissue deoxygenation due to O_2_ extraction.

The NIRS probe (Nimo: Nirox, Brescia, Italy), composed of an emitter and detector pair, was firmly placed on the skin over the lower third of the right *vastus lateralis* muscle (∼10 cm above the proximal border of patella and 3 cm lateral to the midline of the thigh), and secured with a small belt of Velcro straps. Elastic bandages were put around the muscle probe to prevent contamination from localised light. Pen-marks were made over the skin to indicate the margins of the plastic spacer in order to check for any downward sliding of the probe during running. Once secured in place, no sliding of the probe was detected. According to the Monte Carlo method of simulation, based on skin and muscle scattering and absorption characteristics for NIR light, in in-vivo measurements, a source-detector spacing of 20 mm is enough for the NIR light passing through the muscle layer, even when the adipose tissue thickness is 15 mm. Skinfold thickness at the site of application of the NIRS probe was determined before the exercise protocol by a calliper (C10 Plicometer Tanner – Whitehouse; Holtain, Ltd., Crymych, UK): the measured average values of skin and subcutaneous thigh tissue thickness in children with ALL and CTRL were 11.0±1.0 *vs* 9.9±0.9 mm, respectively and not statistically different. Statistically significant correlation between skinfold thickness and muscle O_2_ extraction did not hold for both children with ALL and CTRL (slope −6.10, r^2^ 0.10 and p = 0.65 and slope −6.33, r^2^ 0.02 and p = 0.68, respectively). The probe was connected to a personal computer for data acquisition, A/D conversion, and subsequent analysis. Data were recorded at 2 Hz. Muscle Δ[HHb] data were expressed as a percentage of the maximal deoxygenation reference point obtained by a post exercise leg-cuff ischemia technique (Δ[HHb]/Δ[HHb]isch). Ischemia was obtained by inflating a cuff at 150–250 mmHg (depending on the child's BMI) for 2–3 minutes while children were sitting on the medical bed. The final reading was taken when detection of simultaneous increase of Δ[HHb] and corresponding decrease in Δ[O_2_Hb] reached a plateau.

### Statistical analysis

We applied the Shapiro-Wilk test for normality. For normal distributions we provided mean ± standard deviation (SD) and used Student t test to estimate statistical significant difference between groups. The level of significance was set at *p*<0.05. Regression and correlation analyses were performed using the least squared residuals method. All statistical analyses were performed by utilizing a commercially available software package (Prism 4.0: GraphPad, San Diego, California).

## Results

**Figure 1:**
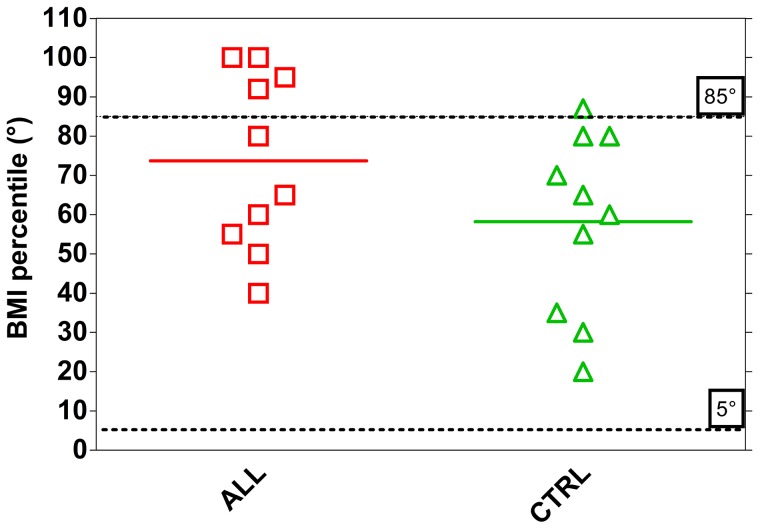
Individual and mean values of body mass index percentile (BMI) in ALL (n = 10) and CTRL (n = 10) children. Statistical significance level was defined as p<0.05.

Based on Shapiro-Wilk test, we found that for both groups the data were drawn from normally distributed populations at the 0.05 level of significance. We therefore evaluated the significance of the differences between groups by Student t test. The mean BMI was 18.4±3.35 kg (m^2^)^−1^ in children with ALL and was not significantly different from CTRL (16.3±1.60). Nevertheless 40% of children with ALL had a percentile BMI>85^th^ while only 1 children in the CTRL group fell in this range (10%) (Figure 1).

Table 2 presents the mean (± SD) basal and peak values of the main variables obtained at rest and at the end of the exercise. The only apparent difference between CTRL and ALL groups concerned the Δ[HHb]_peak_ values: on average, at peak speed, O_2_ extraction in children with ALL was 50% lower than in CTRL, the difference being highly significant (p = 0.018). The coefficient of variation was 55% higher in children with ALL than in CTRL.

**Table 2:** Mean±SD of basal and peak values (with coefficient of variation) of the investigated variables in the two groups of children.

	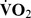	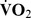	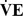 (BTPS)	Pet O_2_	Pet CO_2_	SaO_2_	Δ[HHb]
	(L*min^−1^)	(mL*kg^−1^ *min^−1^)	(L*min^−1^)	mmHg	mmHg		% of ischemia
**CTRL**							
***baseline values***							
mean	0.15	5.91	7.24	105.99	35.34	97.21	\
*SD*	*0.03*	*0.84*	*1.27*	*3.34*	*2.63*	*1.71*	\
***peak values***							
mean	1.06	40.54	38.90	109.78	35.73	95.70	30.40
*SD*	*0.34*	*8.40*	*12.85*	*4.56*	*3.96*	*2.32*	*11.90*
*coef.var.(%)*	*31.89*	*20.71*	*33.03*	*4.15*	*11.08*	*2.43*	*39.14*
**Children with ALL**							
***baseline values***							
mean	0.20	6.48	8.72	106.73	32.69	97.46	\
*SD*	*0.08*	*1.05*	*3.20*	*6.12*	*3.75*	*1.53*	*\*
***peak values***							
mean	1.13	37.02	37.00	109.07	36.58	96.03	15.32*
*SD*	*0.37*	*4.71*	*9.15*	*2.18*	*1.77*	*3.01*	*13.30*
*coef.var.(%)*	*32.63*	*12.73*	*24.74*	*2.00*	*4.84*	*3.13*	*86.70*

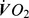
 *: O2 uptake;* 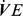 *: pulmonary ventilation; PetO2: pulmonary end tidal of O2; Pet CO2: pulmonary end tidal of CO2; Sa O2: arterial blood saturation; Δ[HHb]: skeletal muscle O2 extraction.*

* p<0.05 significantly different from CTRL peak values.

**Figure 2:**
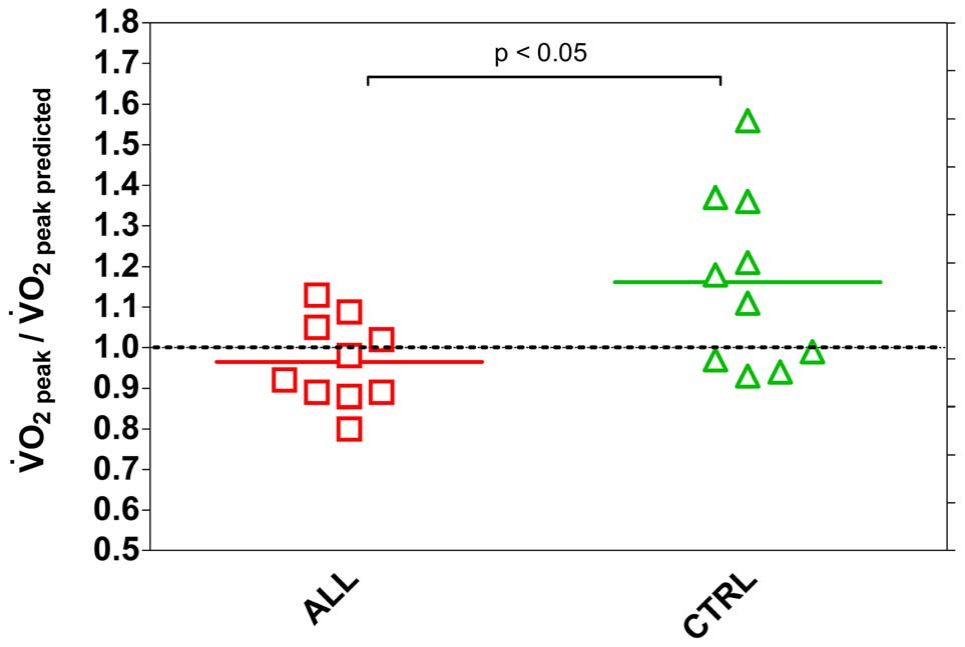
Individual and mean values of 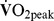 relative to predicted 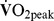 in ALL (n = 10) and CTRL (n = 10) children. Statistical significance level was defined as p<0.05.

The average 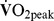, relative to predicted value [23], was about 10% lower - although this was not significant - in children with ALL compared to the CTRL. For CTRL children this ratio was higher than 1 (1.12±0.20) and was statistically greater compared to the ALL group (0.95±0.09). Additionally, the coefficient of variation for children with ALL was remarkably lower than in CTRL (10.2 *vs* 18.2%, respectively), as showed also in Figure 2. Table 2 also shows that no difference in 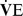, PetO_2_, PetCO_2_, and SaO_2_ was noticed between the children with ALL and CTRL.

The RPE, evaluated by Borg's scale at peak exercise, was not significantly different for children with ALL (16.1±2.4) compared to CTRL (14.5±4.1), the coefficient of variation being 48% lower for children with ALL. The r^2^ values of the correlations between HR and BORG values throughout the execution of the test, were respectively 0.46 (p<0.0001) for ALL and 0.13 (p<0.008) for CTRL.

The average peak workload was the same for children with ALL and CTRL. The treadmill inclination was 15.0±0.9 *vs* 15.2±1.8% and velocity 4.4±0.2 *vs* 4.4±0.5 km h^−1^, respectively and not statistically different. The peak external mechanical work was 56.30±6.35 *vs* 53.48±6.14 watt respectively and was not significantly different in children with ALL *vs* CTRL. The slopes of the 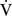
O_2_ *vs* workload linear response, taken as an index of the energy cost of locomotion, were not significantly different between the 2 groups (12.2±1.1 ml O_2_ min^−1^ watt^−1^ in children with ALL, *vs* 15.6±0.7 ml O_2_ min^−1^ watt^−1^ in CTRL, comparison of slopes by covariance analysis p>0.05).

**Figure 3:**
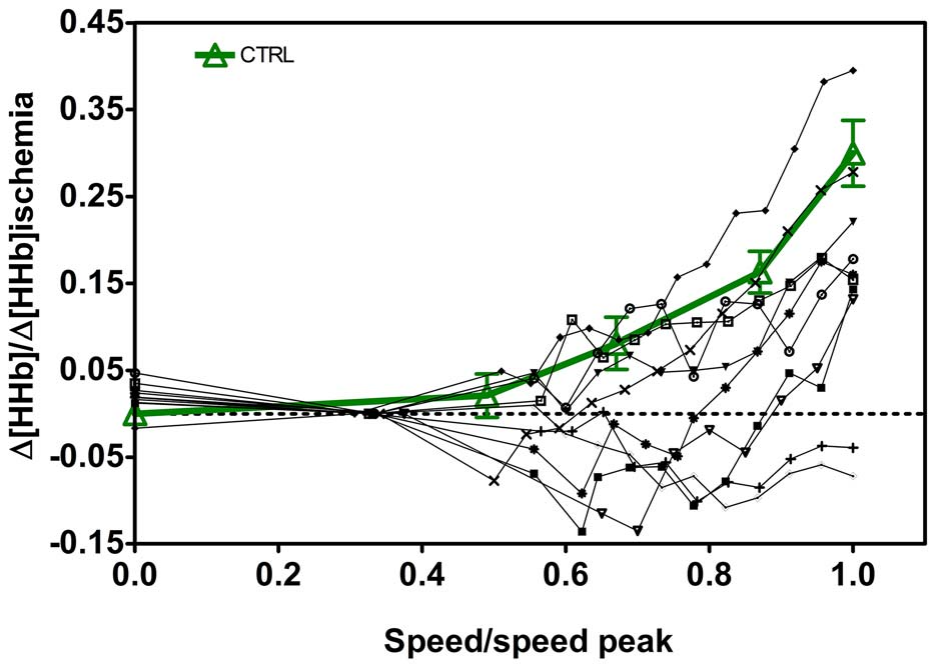
Individual skeletal muscle O_2_ extraction, Δ[HHb], expressed as the ratio of the values obtained during limb ischemia, are presented for children with ALL as a function of speed. CTRL values (thick line) are shown as mean±SE. Speed is expressed relative to peak value. Statistical significance level was defined as p<0.05.

Individual data for O_2_ extraction at the skeletal muscle level, as measured by NIRS and expressed as the fraction of the values obtained during limb ischemia, are presented in Figure 3 for children with ALL as a function of speed, along with the mean (± SE) values obtained in CTRL. The speed values are expressed relative to peak speed, to allow a comparison of the muscle extraction values of O_2_ obtained at the same relative velocity among individuals with varying exercise capacity. Children with ALL present a high variability in terms of O_2_ extraction, ranging from normal to severe deficiency in O_2_ extraction (2 cases).

**Figure 4:**
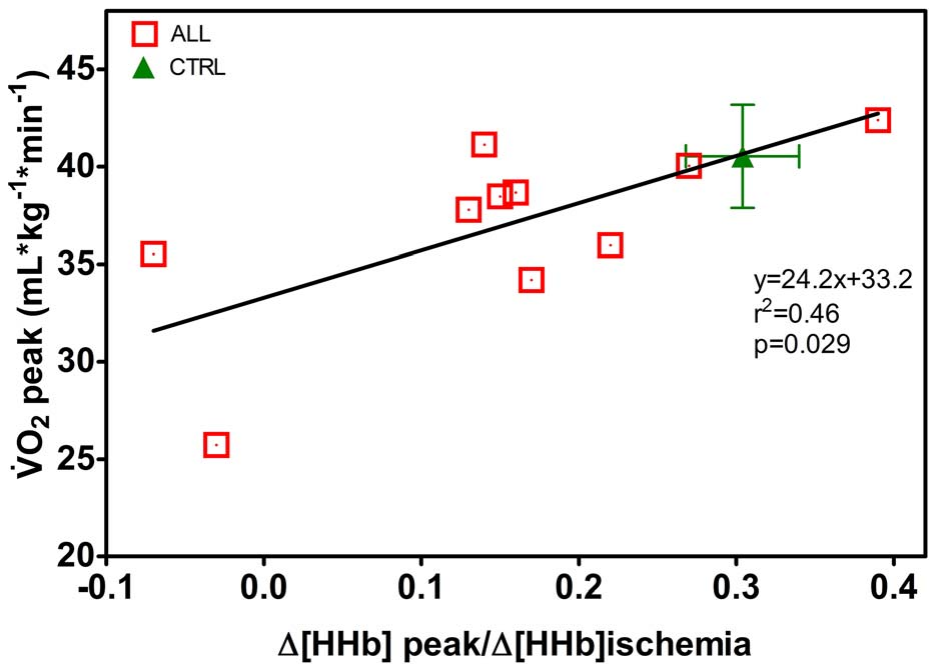
Regression between 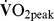 and skeletal muscle O_2_ extraction at peak of exercise, Δ[HHb] _peak_, expressed as the ratio of the values obtained during limb ischemia for children with ALL (n = 10). CTRL values are shown as mean±SE. Statistical significance level was defined as p<0.05.

**Figure 5:**
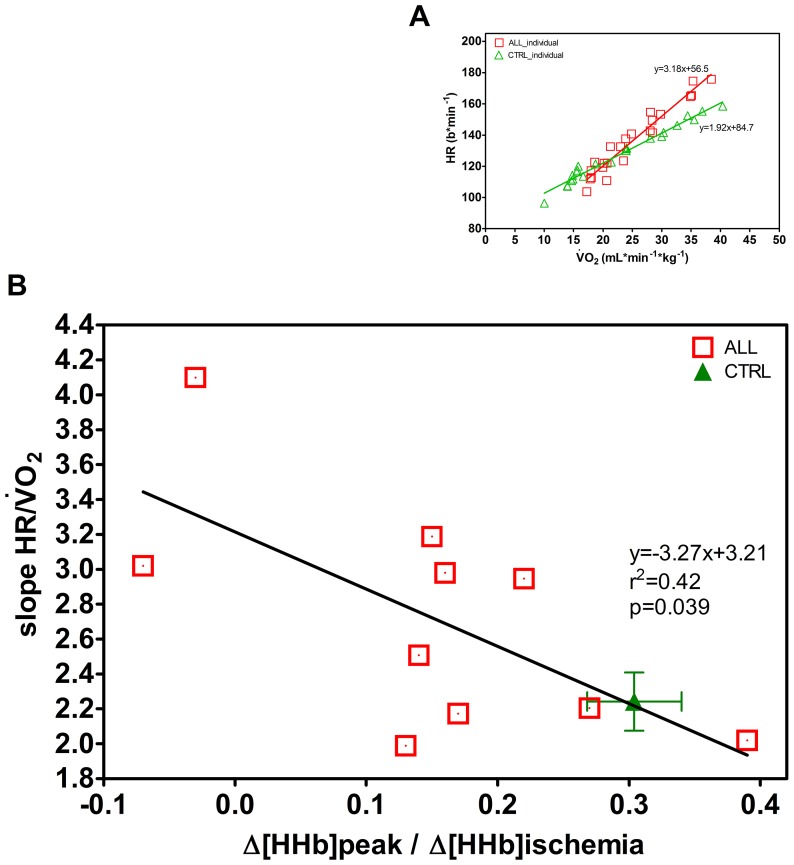
Individual regression for heart rate (HR) *vs* the corresponding 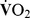 for one ALL and one CTRL child (A graph). Regression between the individual slopes of the HR *vs* 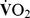 relationship, obtained in children with ALL (n = 10) and skeletal muscle O_2_ extraction at peak of exercise Δ[HHb]_peak_, expressed as the ratio of the values obtained during limb ischemia with the corresponding regression line (B graph). CTRL values are given as mean±SE. Statistical significance level was defined as p<0.05.

In Figure 4, a statistically significant linear regression was found in children with ALL between 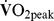 and Δ[HHb]_peak_, note that the value of r^2^ reveals that essentially only about 50% of the variance of 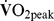 is explained by Δ[HHb]_peak_ as independent variable. No such correlation did hold for CTRL (slope −23.2, r^2^ 0.09 and p = 0.41) so only the average value of 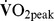
and corresponding Δ[HHb]_peak_ is reported in the Figure. Figure 5 (A) shows, as an example, that the HR *vs* 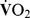 response during exercise was displaced upward and displayed a greater slope on comparing an ALL to a CTRL. Figure 5 (B) shows that for children with ALL there was indeed a statistically significant linear regression with a negative slope between the individual slopes of the HR *vs* O_2_ correlation and muscle O_2_ extraction. Therefore this regression reveals that a greater cardiac response occurs the lower is the peripheral O_2_ extraction capacity. Such correlation was not found for CTRL (slope 1.21; r^2^ 0.08 and p = 0.42) and therefore we only present an average value of slopes of the HR *vs* O_2_ correlation and muscle O_2_ extraction.

The mean 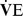 *vs* 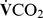 slope in children with ALL was 27.1±2.92 and was not significantly different from CTRL (28.4±2.78).

## Discussion

It has been suggested but not demonstrated that there is O_2_ extraction impairment in children with ALL [5]. This is the first study, to our knowledge, to perform measurements of skeletal muscle O_2_ extraction by NIRS in children with ALL during exercise. This technique was previously adopted by different authors as a non-invasive approach in clinical practice, even in the case of severe diseases, to demonstrate a potential impairment in the O_2_ extraction capabilities of skeletal muscle during exercise [20], [24], [25]. A further novelty of this study is that we provide data on the peripheral O_2_ extraction 1 year on average after the end of malignancy treatment.

The present findings indicate that muscle O_2_ extraction ability during exercise was about 50% lower in ALL, as compared to CTRL, revealing an impairment of various degree in microvascular perfusion and/or the O_2_ shuttle system. Figure 2 shows that an impairment in oxidative muscle extraction could be detected in children with ALL even at submaximal running speeds compared to the CTRL. The same is true at peak workout. In particular, in 2 children with ALL (1 MRD-SR and 1 MRD-IR) O_2_ muscle extraction was so low, at submaximal and maximal exercise rates, as to raise suspicions about an underlying metabolic myopathy. A similar observation was reported in severe disease conditions, such as after heart transplant and in metabolic myopathy, supporting the relevance of the peripheral component in limiting the increase in O_2_ consumption [24]–[26]. Marchese et al. [24] reported a decreased skeletal muscle function in children with ALL early in their course of treatment, with the weakest muscle strength occurring on the 28^th^ day of the delayed intensification phase. Murat et al. [17] found reliable data indicating that muscle weakness is a relevant clinical manifestation in patient with ALL also in the maintenance phase of ALL therapy. Our present data are consistent with the idea that impaired muscle function could be a relevant manifestation in children with ALL and further extend this notion to children after 1 year from the end of malignancy treatment. A limitation of the capacity of muscle fibres to consume O_2_ in leukaemia survivors may be explained at least in part by muscle atrophy due to the catabolic effects of malignancy treatment such as vincristine or corticosteroids. Glucocorticoids inhibit overall protein synthesis in skeletal muscle causing muscle atrophy and loss of muscle function, the effect of corticosteroids being to blunt the stimulatory effect of insulin and amino acids on protein synthesis [14], [15]. Also, the muscle connective tissue undergoes a gene suppression leading to a collagen degradation, especially in fast muscles [14]. There may also be an altered metabolic function of muscle fibres, as in metabolic myopathy, due to immunosuppressive therapy (particularly in female patients). The muscle weakness due to oxidative inefficiency may be subnormal for many years after therapy for childhood leukemia [27], [28]. An additional explanation is that muscle atrophy and altered muscle function may be further worsened by bed rest and sedentary habits [5]. Although aerobic activities could prevent muscle atrophy, exercise may not to be able to compensate for the changes observed during corticosteroid treatment [15]. As a result, when physical exercise is kept low, muscle atrophy and early fatigue become self-perpetuating conditions [6]. To compensate for these deficiencies, the possible benefits of prophylactic and individually planned exercise should be studied [28].

A mild average difference in relative 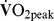 – which is however not statistically significant – was seen when comparing children with ALL with CTRL, indicating a reduced exercise capacity only in some children with ALL. This finding is at variance with data from van Brussels et al. [5] meta analysis, based on a systematic review of the literature, indicating a reduced 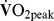 in children with ALL. However, some heterogeneity among the studies considered was admitted by van Brussels et al. [5] in their meta analysis: Vizinova et al. [29] and Hauser et al. [30] did not find significant differences between ALL survivors and CTRL participants, the possible explanation residing in differences in the level of physical activity. Also in our study an expected grade of variability in level of aerobic fitness was shown by children with ALL, due to the fact that ALL survivors were vigorously encouraged to be physically active by the oncologists (as were demonstrated by the ratios of 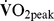 to predicted 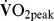 near 100% in more than half of the cases). Additionally, the most important difference in previous studies regarding a decrease in 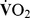 in children with ALL, as compared with findings in our study, relates to the lapse of time between the end of the therapy and the date of the functional evaluation. Our study was performed within 1 year on average from the end of malignancy treatment, while in other studies the functional evaluation was done over a period extending up to 5 years after the end of therapy. It appears difficult to predict how sedentary habits might affect exercise capacity after such a long time, during the crucial teenage years both in children with ALL and CTRL.

In healthy people, the maximum cardiac output rather than the maximal rate of tissue O_2_ extraction, is the main factor limiting the 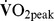 for exercise involving large muscle mass,. The same phenomenon is confirmed by McNarry et al. [31] in a population of healthy girls, suggesting the same physiological responses in children. We confirm this same pattern in our CRTL. However, in children with ALL there was a tendency to show a greater increase in HR with increasing 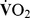 when the muscle O_2_ extraction was reduced. The limitation in 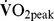 children with ALL may reflect both a decrease in muscle O_2_ extraction (Figure 4) and/or a decrease in O_2_ delivery [Figure 5). Considering HR as a proxy of muscle blood flow (and presumably O_2_ delivery) in some children with ALL there was a compensatory behaviour of the central component, in order to increase convective O_2_ delivery to the muscle. Our data showed a decrease in muscle oxygen extraction as well as a greater slope of the HR vs 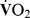 relationship. Interestingly, the strongest cardiac response occurs in children with the lowest extraction capacity at skeletal muscle level. Whereas higher HR values for the same 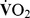 are generally considered a sign of poor exercise capacity, the leftward shift with a higher slope of regression in the children with ALL reveals an enhanced cardiovascular response for a given metabolic response (Figure 5 A). Note, that compared to a normal O_2_ extraction seen in CTRL at peak of exercise, 70% of children with ALL showed an extraction rate at least 30% lower than CTRL. The negative correlation between muscle O_2_ extraction and the slopes of the HR *vs* 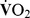 relationships has been previously reported for patients with metabolic myopathies [20]. Thus, contrary to what happens with healthy participants, in some children with ALL, the decreased muscle O_2_ extraction capacity that seriously limits 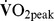 may reflect a state of myopathy possibly due to corticosteroids treatment, and/or to sedentary life habits [5], [16]. A potential correlation between the severity of disease, as based on the MRD risk, and our functional variables, namely the slope of the HR vs VO_2_ relationship and muscle O_2_ extraction (Figure 5 B) appears critical. Indeed in 2 children with ALL categorized as MRD-S and MRD-I risk, the 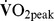 was equally strongly decreased, and the highest cardiac response reflected an extreme reduction of oxygen muscle capacity. Conversely in 1 child graded as MRD-H, the 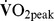 was essentially normal, the extraction was partially maintained and higher than in MRD-S and MRD-I children, while the cardiac response was essentially normal. We therefore conclude that in our study the severity of MRD is poorly correlated to the potential limitation of the O_2_ transport and utilisation chain.

The RPE, estimated using the Borg Scale, is not commonly used among healthy children and in fact a great coefficient of variation was found in CTRL. Interestingly, children with ALL were able to rate perceived exertion in a real convincing way as the index of fatigue correlated persuasively with the increase in HR values. Our personal consideration is that, probably, children with ALL are more used to the experience of fatigue or uneasiness and therefore can rate it better as a subjective feeling.

Excessive fatness is another reported consequence of malignancy treatment in children with ALL. This has been related to cranial irradiation or growth hormone (GH) insufficiency and to the cumulative doses of anthracyclines or corticosteroids, as well as to the type of corticosteroid used [32]. Our children with ALL do not have a reported GH insufficiency and only 1 child received cranial irradiation because of MRD-HR, so that a greater proportion of ALL participants with a BMI greater than 85^th^ percentile, might again be attributed to their sedentary habits and/or corticosteroid use. Although short-term exposure to glucocorticoids is not associated with coronary diseases during childhood, it is of interest that known cardiovascular risk factors (abdominal adiposity and insulin resistance) are increased even after a short treatment, and the magnitude of the changes induced is proportional to the dosage of the drug [33]. Long-term corticosteroids effects, and the small amount of leisure-time dedicated to physical activity could be a hard inheritance for leukemia survivors reaching adult age. In fact, as confirmed by a recent study, survivors of leukemia were more likely than the general population to report no leisure-time physical activity, combined with unhealthy diet habits, contributing to altered body composition parameters in adulthood [6].

Children with ALL were all subjected to the same treatment, receiving standard doses of cardiotoxic drugs, such as anthracyclines (<300 mg (m^2^)^−1^, and none was subjected to thoracic irradiation. A study performed by our Department of Paediatrics shows that in the absence of any signs or symptoms of heart failure, female ALL survivors treated with low cumulative anthracycline doses showed a reduced left ventricular mass and wall thickness [34]. It should also be pointed out that we consider HR merely as a substitute for O_2_ delivery, but we are anyway inclined to believe that impaired cardiac function is responsible, at least partly, for the reduced exercise capacity of some of our children with ALL. According to van Brussel et al. [5], even more interestingly, a greater coefficient of variation in 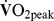 and of the ratio of 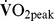 to predicted 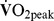 in CTRL children may be interpreted as resulting from a variable state of fitness (where children followed either a sedentary lifestyle or were engaged in recreational activities). Conversely, a lower coefficient of variation in children with ALL may reflect a more homogeneous lifestyle reflecting sedentary habits only as compared to their healthy peers.

SaO_2_ during exercise, an index of efficiency of the central component in the O_2_ transport and utilization chain from the lung to tissues, remained normal in our children with ALL. Also PetCO_2_ values and the slope of 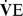 *vs* 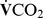, which are not different in children with ALL compared with CTRL, indicate normal lung diffusion function and appropriate ventilatory efficiency during exercise [22], [25]. This finding is to some extent in conflict with the reported impairments in lung function [9] due to the late effects of MTX and cyclophosphamide in children aged 14.6 years (which is older than our children, who are mostly pre-pubertal). However in Jenney et al. study [9], the evaluation was performed more than 4 years after the end of therapy (which is later than our children, who were evaluated 1 year after the end of malignancy treatment). In fact, prolonged immunosuppression during maintenance chemotherapy may lead to an increased frequency of lower respiratory tract infections. It is indeed known that children who suffer from acute respiratory diseases in early life (even without immunosuppression) may develop more chronic problems as they get older [9].

## Conclusions

The present study shows that the NIRS technique allows us to identify large inter-individual differences in levels of impairment in muscle O_2_ extraction in children with ALL. The outcome of these findings is variable and may reflect either muscle atrophy due to lack of use or, in the most severe cases, an undiagnosed myopathy. Our data support the argument that the impaired O_2_ extraction in children with ALL may be partly compensated by a central component (i.e. a greater increase in HR when increasing 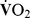). Compensation reflects the balance between the extent of the impairment on peripheral O_2_ extraction and the functional condition of the heart. This multifaceted interaction may justify controversies about the 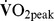 reported by different research groups [5]. Separating out the effects of cancer and treatment between central and peripheral components of the O_2_ delivery chain should be of interest to clinicians for longitudinal evaluation of potential functional impairment in order to set appropriate individually tailored training/rehabilitation programmes [35]–[38]. The long-term objective of the cure and care of children with cancer is that they become resilient, efficient and autonomous adults with a high health-related quality of life.
